# Surface ocean warming and acidification driven by rapid carbon release precedes Paleocene-Eocene Thermal Maximum

**DOI:** 10.1126/sciadv.abg1025

**Published:** 2022-03-16

**Authors:** Tali L. Babila, Donald E. Penman, Christopher D. Standish, Monika Doubrawa, Timothy J. Bralower, Marci M. Robinson, Jean M. Self-Trail, Robert P. Speijer, Peter Stassen, Gavin L. Foster, James C. Zachos

**Affiliations:** 1 School of Ocean and Earth Science, University of Southampton Waterfront Campus, National Oceanography Centre, Southampton, UK.; 2 Department of Earth and Planetary Sciences, University of California Santa Cruz, Santa Cruz, CA, USA.; 3 Department of Geosciences, Utah State University, Logan, UT, USA.; 4 Department of Earth and Environmental Sciences, KU Leuven, Leuven, Belgium.; 5 Department of Geosciences, Pennsylvania State University, University Park, PA, USA.; 6 Florence Bascom Geoscience Center, U.S. Geological Survey, Reston, VA, USA.; 7 Directorate Earth and History of Life, Royal Belgian Institute of Natural Sciences, Brussels, Belgium.

## Abstract

The Paleocene-Eocene Thermal Maximum (PETM) is recognized by a major negative carbon isotope (δ^13^C) excursion (CIE) signifying an injection of isotopically light carbon into exogenic reservoirs, the mass, source, and tempo of which continue to be debated. Evidence of a transient precursor carbon release(s) has been identified in a few localities, although it remains equivocal whether there is a global signal. Here, we present foraminiferal δ^13^C records from a marine continental margin section, which reveal a 1.0 to 1.5‰ negative pre-onset excursion (POE), and concomitant rise in sea surface temperature of at least 2°C and a decline in ocean pH. The recovery of both δ^13^C and pH before the CIE onset and apparent absence of a POE in deep-sea records suggests a rapid (< ocean mixing time scales) carbon release, followed by recovery driven by deep-sea mixing. Carbon released during the POE is therefore likely more similar to ongoing anthropogenic emissions in mass and rate than the main CIE.

## Teaser

Geochemical records suggest a global precursor carbon cycle event drove ocean warming and acidification before the PETM.

## INTRODUCTION

Geologically abrupt [<10 thousand years (ka)] carbon perturbations, such as the Paleocene-Eocene Thermal Maximum (PETM, ~56 million years ago), are useful natural experiments to examine the Earth system’s response to rapid carbon invasion (*1*, *2*). A key feature of the PETM is a negative carbon isotope excursion (CIE) of 3 to 6‰ observed globally in sedimentary records that is interpreted as the result of a large injection of isotopically light carbon into atmosphere-ocean reservoirs (*3*). The biogeochemical and environmental responses included ocean warming, deoxygenation, ocean acidification, sea-level rise, and an enhanced hydrologic cycle (*4*–*8*). However, determining the succession of environmental changes at the CIE onset in deep-sea cores, which typically provide the most continuous deposition, is challenging as most sections lack calcareous microfossils, are highly condensed or truncated as a result of severe chemical erosion (*9*, *10*), and/or are complicated by bioturbation, reworking, and winnowing. The uncertainty surrounding the magnitude, pattern, and rate of carbon release therefore hinders efforts to identify the source and causal mechanism, and thus, the role of feedbacks in driving the warming observed during the initial stage of the PETM remains debated (*11*, *12*).

### Environmental precursors to the PETM

Paleoceanographic records indicate both gradual long- and short-term warming before the CIE onset, which are consistent with a climatic threshold being crossed by either rising atmospheric carbon dioxide (CO_2_) levels or orbital drivers (*12*–*14*). In this context, a precursor warming could be viewed as an initial sign of increasing instability of a thermally sensitive carbon source such as continental margin methane hydrates, and a potentially important positive feedback mechanism to sustain the subsequent warming (*12*, *15*). In either case, the early warming is thought to be driven by greenhouse gases, presumably CO_2_, although δ^13^C or other geochemical records fail to record such an injection of carbon before the main CIE (*6*, *11*, *16*, *17*). Several arguments have been put forward to explain the observed early warming and apparent lack of δ^13^C change in sedimentary records, which include an injection of carbon with a δ^13^C composition close enough to 0‰ insufficient to alter the exogenic carbon pool (*12*, *13*). An alternative explanation is that current carbon cycle proxy records are too poorly resolved or preserved, particularly during the PETM onset, to capture a transient precursor event. Terrestrial pedogenic carbonate-based carbon isotope records, however, clearly document two distinct δ^13^C excursions, herein following Bowen *et al.* (*18*) termed the pre-onset excursion (POE) and CIE, reflecting multiple carbon injections associated with the PETM onset (*18*). A few shallow marine carbon isotope records also capture dual δ^13^C excursions (*13*, *19*–*21*), but the older excursion is often relatively small, possibly due to truncation. Most of the marine POE records are based on δ^13^C analyses of bulk sediment samples, which represent a heterogeneous isotopic signal and so are not strictly indicative of a marine dissolved inorganic carbon (DIC) signature. Hence, it remains uncertain whether the POE is a global event and, ultimately, whether it relates to a carbon source common to the main CIE itself.

**Fig. 1.**
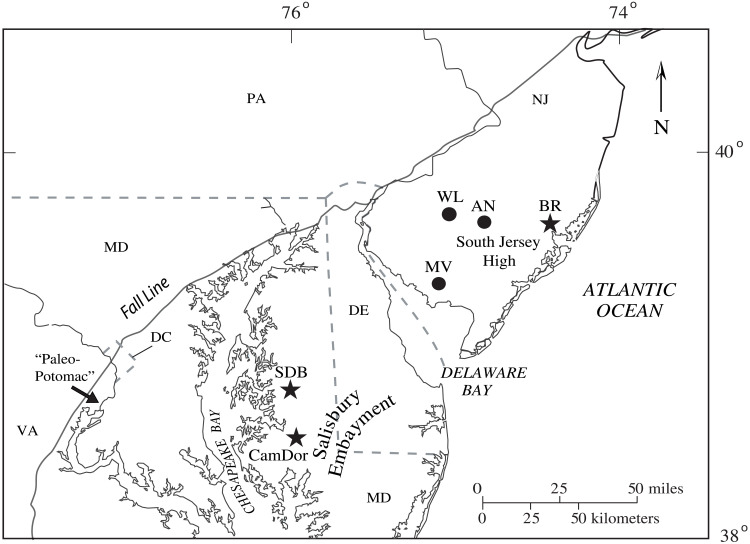
Regional map of core drilled within the Mid-Atlantic Coastal Plain [modified from (*19*)]. Trace element, stable isotope, and boron isotope data presented here from Bass River (BR), Cambridge-Dorchester (CamDor), and SDB are marked by star symbols. Core holes Ancora (AN), Millville (MV), and Wilson Lake (WL) discussed in this study are marked by circle symbols.

If the POE event was global and short-lived, occurring in less than several thousand years as estimated by Bowen *et al.* (*18*), in theory the chemical signal would be largely limited to atmosphere-surface ocean carbon reservoirs and should include evidence of surface ocean acidification. The likelihood of capturing such a short-lived event in deep-sea sections, however, would be remote, particularly in the interval directly preceding the PETM given the widespread deep-sea carbonate sediment dissolution horizon at this time (*9*, *10*). Instead of using deep-sea sections, we focus here on shallow marine sections cored by the U.S. Geological Survey at South Dover Bridge (SDB) and Cambridge-Dorchester Airport (CamDor) located in the Maryland Coastal Plain of the Salisbury Embayment (Fig. 1) (*19*). Faunal assemblage data indicate deposition at SDB in deep middle to outer neritic environments during the PETM (*22*, *23*). Maryland marine siliciclastic shelf facies during the PETM are characterized by exceptionally high sedimentation rates and less dissolution of carbonate than in deep-sea sections (*24*). The Mid-Atlantic Coastal Plain marine siliciclastic shelf sequences offer the best opportunity to capture and resolve the details of short-lived events, especially given that uppermost Paleocene sediments are removed or condensed in the deep sea by carbonate dissolution associated with PETM ocean acidification (*9*, *10*). The main disadvantage is that shallow shelf deposition, while fast, is often episodic or discontinuous. Furthermore, higher siliciclastic fluxes diluted marine sediments and result in sparsely available calcareous microfossils, although some well-preserved foraminifera specimens are present (*25*, *26*). Foraminiferal stable isotope (δ^18^O and δ^13^C) and trace element to calcium ratios (Mg/Ca and B/Ca) were generated here to reconstruct surface ocean temperature and carbonate chemistry. However, the low foraminiferal abundance limits the applicability in marine shelf settings of the boron isotope-pH proxy that previously provided a number of insights concerning carbon cycle changes at the CIE onset (*6*, *11*, *17*, *27*). To overcome the prohibitive sample size requirements of the traditional analytical methods (*28*), we use a novel laser ablation approach (*29*) to analyze the boron isotope (δ^11^B) of individual benthic foraminifera to reconstruct seawater pH at unprecedented resolution for deep-time marine sedimentary archives. We use these geochemical records along with a numerical carbon cycle model, LOSCAR (*30*), to provide initial constraints on the tempo and magnitude of carbon released during the POE and the PETM onset.

## RESULTS

### Maryland PETM stable isotope and trace element anomalies

**Fig. 2.**
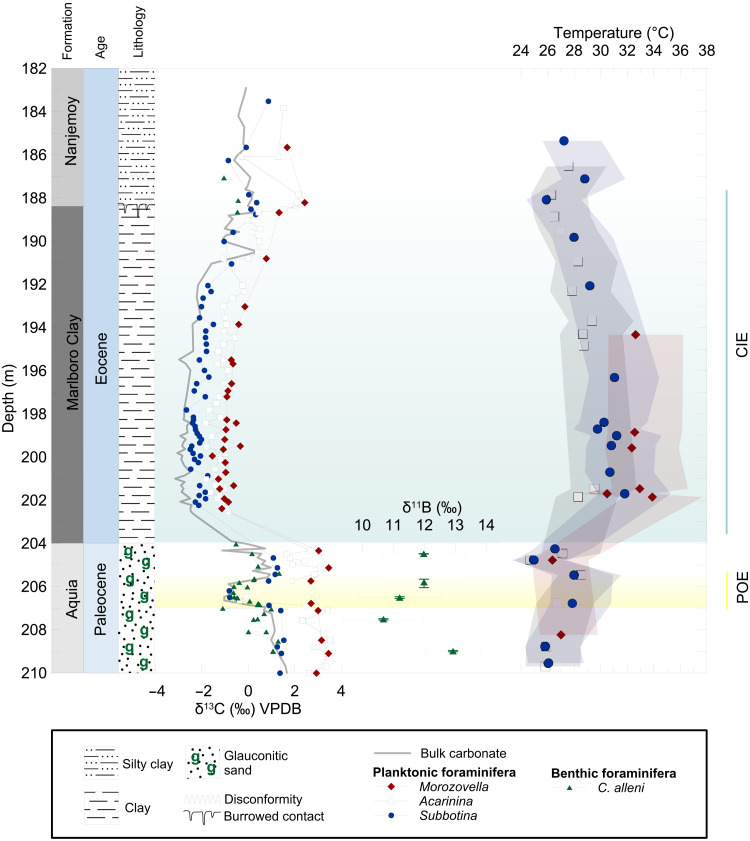
SDB carbon isotope (δ^13^C) bulk carbonate, planktonic, and benthic foraminifera records. Planktonic foraminifera Mg/Ca estimated ocean temperatures, and error envelope includes uncertainty in Mg/Ca seawater composition. Average δ^11^B of single specimen benthic foraminifera (*C. alleni*), with 2 SE associated with the mean for each depth and the uncertainty in our internal reference material PS69/318-1. Vertical error bars of δ^11^B represent the full sample depth range of the multiple individual measurements represented by the average δ^11^B value. Highlighted in yellow is the POE and in blue is the body of the CIE.

Bulk carbonate δ^13^C records from Maryland (SDB and CamDor) exhibit a ~4 to 6‰ negative CIE at the Paleocene-Eocene boundary typical of the siliciclastic sequences (e.g., Marlboro Clay) of the continental shelf throughout the Mid-Atlantic Coastal Plain (Fig. 1) (*26*, *31*, *32*). Associated with the CIE, documented in all planktonic foraminiferal genera is a ~1.0‰ δ^18^O negative shift, and a 40% decline in B/Ca values (fig. S1) coeval with a 35% increase in Mg/Ca values (fig. S1). At SDB, throughout the duration of the CIE body (204 to 197.82 m), planktonic foraminiferal δ^13^C and δ^18^O values remain depleted and gradually recover toward background Paleocene values in the uppermost part of the section (fig. S1). A burrowed disconformity at the base of the overlying Nanjemoy Formation (188.4 m) in the SDB core confirms a hiatus and incomplete preservation of the CIE recovery (Fig. 2) (*19*). Stable isotope (δ^13^C and δ^18^O) values track ecological depth habitat preferences, with surface dwellers (*Morozovella* and *Acarinina* spp.) recording the most positive δ^13^C and negative δ^18^O values relative to accompanying deeper dwellers *Subbotina* spp. and benthic species *Cibicidoides alleni* (fig. S1). Stable isotope ecological depth hierarchy trends are also observed at nearby New Jersey sites (*26*, *31*, *32*). Following a similar pattern to paired stable isotope records, peak Mg/Ca and minima B/Ca values are reached following the CIE onset and are maintained throughout the duration of the CIE body (fig. S1). Calculation of planktonic foraminifera Mg/Ca-derived temperatures yields average Paleocene temperatures of 26 ± 1.5° to 2.6°C, depending on the estimate of Mg/Ca seawater composition (Fig. 2). For more detailed information on Mg/Ca-derived ocean temperature reconstruction, see the Supplementary Materials. Mg/Ca-based seawater temperatures reach maximum values of 29° to 32°C directly following the CIE onset, similar to planktonic foraminiferal Mg/Ca-based temperatures generated at Bass River core site (*25*). Absolute Mg/Ca and B/Ca values are relatively similar among planktonic foraminiferal genera, indicating a relatively homogeneous upper water column (fig. S1), with the exception of slightly elevated Mg/Ca of *Morozovella* spp. corresponding to 1° to 2°C higher temperatures during the CIE body (Fig. 2). The warmer temperatures recorded by *Morozovella* spp. could be due to shallow calcification depths compared to *Acarinina* spp. or a shift in seasonal blooms (*31*). Planktonic foraminifera Mg/Ca and B/Ca values gradually recover in concert with δ^13^C records, with absolute values approaching pre-event levels (fig. S1).

### Ocean warming and carbon cycle changes

The geochemical data from Maryland (SDB and CamDor) substantially increase the resolution of isotope and trace element records immediately before the CIE onset. The δ^13^C records (bulk carbonate and foraminifera) at SDB and, to a lesser extent, CamDor reveal a distinctive feature with two discrete negative δ^13^C excursions: the main CIE at the PETM onset and another during the latest Paleocene (Fig. 2 and fig. S2). The older CIE, referred to as the POE (207.1 to 205.9 m) (*18*), is characterized at SDB by an abrupt 1.0 to 1.5‰ negative shift observed in surface (*Acarinina* spp.) and deeper (*Subbotina* spp.) dwelling planktonic foraminifera and in benthic foraminifera (*C. alleni*) (Fig. 2). Minimum planktonic foraminiferal δ^13^C values are recorded within the plateau of low values defined in the bulk carbonate δ^13^C record. In general, the δ^13^C benthic foraminiferal record follows complementary bulk and planktonic foraminiferal records, with the exception of several negative values measured below the POE onset. It is possible that some erosional sediment loss occurred at the base of the POE and could explain the discrepancies in δ^13^C structure between bulk and foraminiferal records. Planktonic and benthic foraminiferal δ^13^C values gradually return to pre-POE values in conjunction with the bulk carbonate record. Superimposed on the POE anomaly is an overall background δ^13^C decrease leading up to the CIE onset. The δ^18^O foraminiferal records display small fluctuations of less than 0.5‰ and no apparent shift concurrent with the POE (fig. S1). The lack of a warming signal in SDB δ^18^O foraminifera records is not unexpected as two processes could act to offset a temperature-related δ^18^O decrease. A seasonal increase of local salinity (increasing δ^18^O_sw_) during the period when planktonic foraminifera are blooming or a decline in carbonate saturation (with lower pH) would increase seawater carbonate fractionation, thereby enriching shell δ^18^O, possibly enough to offset the decrease related to rising temperature by approximately 0.25‰/1°C (*33*). A potential pH effect on stable isotopes during the POE would result in higher planktonic foraminiferal δ^18^O and δ^13^C values and dampen the excursions observed (*34*, *35*). *Acarinina* and *Subbotina* spp. B/Ca and Mg/Ca records exhibit similar trends at the POE and CIE, consistent with ocean acidification and warming (fig. S1). However, the magnitude of planktonic foraminiferal B/Ca and Mg/Ca change is nearly 50% smaller at the POE relative to the CIE (fig. S1). Across the POE, surface seawater temperatures warmed by at least ~2°C as derived from Mg/Ca planktonic foraminiferal records and returned to pre-event temperatures immediately before the CIE onset (Fig. 2). Given the coarse resolution of geochemical data records due to low foraminiferal abundances, it is likely that the SDB records only partially capture the extent of warming and acidification.

**Fig. 3.**
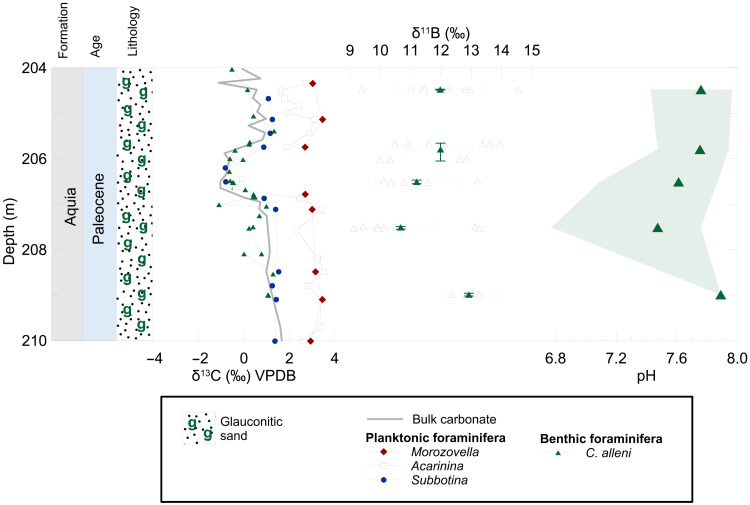
POE interval at SDB carbon isotope (δ^13^C) bulk carbonate, planktonic, and benthic foraminifera records. Single specimen δ^11^B of benthic foraminifera (*C. alleni*) is represented by solid symbols, and the multiple specimen average δ^11^B is represented by open symbols. δ^11^B data presented include 2 SE associated with the mean for each depth and the uncertainty in our internal reference material PS69/318-1. Vertical error bars of δ^11^B represent the full sample depth range of the multiple individual measurements represented by the average δ^11^B value. pH reconstruction based on analysis of average δ^11^B in benthic foraminifera (*C. alleni*). An initial surface Atlantic Ocean pH = 7.89 was assumed, and error uncertainty was propagated using a Monte Carlo approach.

Benthic foraminiferal δ^11^B, based on averages of single analyses of individual specimen (number of individuals = 3 to 8), rapidly declines by 2.1 ± 2.0‰ based on the average δ^11^B difference between the POE versus pre-POE (Fig. 3). The decline in benthic foraminiferal δ^11^B broadly occurs in conjunction with δ^13^C records and surface warming at the POE onset (Figs. 2 and 3). A detailed comparison between δ^13^C and δ^11^B is made difficult by the low resolution of the latter record and the relatively high short-term variability of the former. While it appears that the pH excursion occurs slightly before the POE as defined by the bulk and foraminiferal δ^13^C records, we caution against overinterpreting this feature at this stage. The presence of excursion δ^13^C values before the POE in the benthic record and lack thereof in the δ^13^C bulk carbonate and planktonic foraminifera records could be an artifact of the low sampling resolution combined with size-dependent or taphonomic differences in sediment mixing (*36*). Given the contrasting measurement approaches for δ^11^B and δ^13^C (single foraminifera versus bulk foraminifera, respectively) combined with variations in seasonality, bioturbation and preservation could introduce some bias in timing between the two isotope records. The relative stratigraphic positions of the isotopic excursion onsets should therefore be viewed cautiously with respect to temporal variability. Regardless, the negative δ^11^B excursion at the POE indicates a pH decline possibly comparable to the CIE when compared to global pelagic sites (*6*, *11*, *17*, *27*), Bass River, and Millville (*6*), albeit at lower resolution given the limited availability of foraminiferal material in the New Jersey cores. The determination of the absolute magnitude of the pH excursion at the POE is limited by both the uncertainty of initial environmental conditions and the larger uncertainty associated with laser ablation analyses generated in this study (±~1.0‰ versus 0.2 to 0.3‰ for traditional solution-based methods), which makes determining the absolute magnitude of the pH excursion at the POE rather uncertain. Reconstructing ocean pH using boron isotopes in foraminifera also requires a knowledge of calcification hydrography (e.g., mainly temperature and salinity), appropriate species-specific δ^11^B-pH calibration, and the boron isotopic composition of seawater (δ^11^B_sw_). To address the uncertainty of Paleogene δ^11^B_sw_ on the magnitude of ocean pH (ΔpH), we adopted a Monte Carlo approach used previously by Gutjahr *et al.* (*11*) (see Materials and Methods). A full propagation of uncertainty, however, allows us to determine that the POE pH excursion at the Maryland sites was greater than −0.08, with an upper limit that is poorly constrained but overlaps with the magnitude estimated for the main CIE observed elsewhere (Fig. 3 and table S2).

## DISCUSSION

### Regional environmental trends in the Salisbury Embayment

Several key observations are ubiquitous among Paleocene-Eocene boundary shelf sections within the Salisbury Embayment in the Mid-Atlantic Coastal Plain. Surface ocean warming of 3° to 5°C (derived by Mg/Ca) at the CIE onset is recorded at the SDB (Fig. 2) and Bass River core sites (*25*). Planktonic foraminiferal B/Ca records at SDB are of similar absolute values, magnitude, and trends seen in nearby shelf and pelagic sites, in support of previous findings of near-uniform global surface ocean acidification signal (*6*). The prominent feature of the Maryland core sites is two discrete δ^13^C anomalies associated with the PETM onset (Fig. 2). The uppermost Paleocene POE is defined by anomalous δ^13^C values relative to background records and, along with its recovery, is stratigraphically coherent (Fig. 2). Based on the depth separation between the POE and CIE, the uniform δ^18^O, and a complete sequence of foraminiferal and nannofossil biozones (*23*, *37*), it is clear that the records are temporally continuous and unaltered by sediment mixing or slumping. Following the POE, but before the CIE onset, planktonic foraminifera “excursion taxa” appear in the Salisbury Embayment, suggesting a similar ecological and local environment during the latest Paleocene and PETM (*23*, *38*). The presence of excursion fauna before the CIE, likely in response to early environmental change and warming (*13*), suggests a temporal connection between the POE and the PETM onset. The POE is clearly expressed in bulk δ^13^C records from SDB (*19*) and CamDor (*39*) (fig. S2). Bulk sediment stable isotope records are known to reflect a mixed signal that does not necessarily represent ambient oceanic chemistry but rather the combined environmental, biological, and nonequilibrium isotopic effects. The presence of the POE in both planktonic and benthic foraminiferal records confirms that the SDB δ^13^C signal reflects marine DIC changes. Similarities in the trend and pattern of marine and continental δ^13^C records suggest that the POE was global, as was previously recognized for the main CIE of the PETM (fig. S2) (*40*).

### Constraints on the POE duration

If the continental-marine POE is isochronous, the relative duration of the event should be comparable. The cyclical deposition of the floodplain paleosols in the Big Horn Basin provides well-constrained cyclostratigraphic duration estimates for the POE, CIE, and subsequent Eocene hyperthermals (*18*, *41*). Orbital-driven shifts in hydroclimate combined with rapid subsidence at Big Horn Basin resulted in rhythmic paleosol deposition and high expanded sedimentary sequences spanning the late Paleocene to early Eocene. In contrast, the Mid-Atlantic margin was slowly subsiding, with accommodation space increasing only during periods of eustatic sea-level rise, such as during the CIE, which resulted in comparatively condensed sequence (*7*). Given the coarse resolution of regional stratigraphic age models in upper Paleocene sediments (*32*, *42*) and the character of deposition in shallow marine settings, direct estimation of the POE duration at SDB is unreliable. As is typical of inner shelf facies, deposition was discontinuous and highly episodic (e.g., stochastic), with brief episodes of rapid deposition (*43*) that were followed by erosional/nondepositional intervals, resulting in time being captured in short snapshots with potentially long temporal gaps (*44*). The high spatiotemporal sedimentation variability ensures uneven representation of time, but in theory, short-lived events can be captured, albeit in a small percentage of sections. The Maryland core sites (SDB and CamDor) are most proximal to the primary drainage and delta of the paleo-Potomac River, also referred to as an “Appalachian Amazon” (*45*), and could yield higher temporal sediment fluxes compared to the more distal New Jersey sites, maximizing the likelihood to capture a short-lived event during the latest Paleocene (Fig. 1). This appears to be the case with the POE, where only Maryland core sites SDB and CamDor capture the full POE δ^13^C anomaly (fig. S2), while the New Jersey core sites only partially resolve the event (Wilson Lake and Bass River) (*13*, *25*), or do not capture it at all (Millville, Ancora) (*31*). Unfortunately, the high spatiotemporal deposition along the Mid-Atlantic Coastal Plain coupled with coarse biostratigraphic age control within the Aquia Formation makes correlation between sites and splicing together a continuous composite record challenging. Current late Paleocene sedimentation rates are limited by the lack of stratigraphic tie points and potential episodes of condensation. Assumptions of linear late Paleocene sedimentation rates using regional biostratigraphic constraints yield a POE duration ranging from 40 to over 200 ka (table S1 and excluding the wide variance in estimates of sedimentation rates at Wilson Lake), and roughly 100 and 350 ka between the POE base and CIE onset, which is two orders of magnitude longer than the duration previously estimated at Big Horn Basin as less than 2 ka (*18*).

If the POE events in marine and terrestrial records are isochronous, sedimentation rates during the POE at SDB need to have been 20-fold higher than the current average estimates for the Aquia Formation (table S1). It must be noted that the POE interval at SDB consists of more fine-grained sediments compared to the background Paleocene sediments (*19*) and thus hinting toward a temporal sediment flux increase from the paleo-Potomac River system. These episodic and highly variable sedimentation rates are entirely consistent with shelf depositional models, and a comparable regional increase in sediment flux has occurred during the PETM onset in the basin. Based on the depositional setting of the Salisbury Embayment and δ^13^C excursion preserved in a small percentage of shelf sites, we must assume that the POE spans a few centuries or possibly several millennia at most.

In addition to the above sedimentological arguments, the short-lived nature of the δ^13^C excursion and modest degree of ocean warming associated with the POE are difficult to reconcile, with a large carbon input and emission duration longer than ocean mixing time scales, at most several thousand years. Existing box model simulations are only able to match the δ^13^C recovery between the POE and CIE when the POE duration is less than ~2 ka, carbon is injected into the atmosphere, and there is a slowdown of surface–deep ocean mixing (*18*). In such a scenario, the rapid recovery of atmosphere and surface ocean CO_2_/pH, temperature, and δ^13^C simply reflects the mixing of carbon into the much larger deep ocean DIC reservoir. A prolonged duration would necessarily involve the deep-ocean carbon reservoir and require long-term geologic feedbacks like silicate weathering in order for the carbon cycle to completely recover (*18*). If we consider the lack of a deep-sea isotopic expression of the POE event at face value, it suggests that the duration and mass of carbon release was insufficient to alter the deep-ocean reservoir. The lack of a deep-sea POE event is consistent with a lack of a negative excursion before the CIE in deep-sea benthic records or indirect evidence for deep-sea acidification as seafloor carbonate dissolution before the main CIE (*46*). While chemical erosion associated with the CIE might have erased evidence of a precursor event in carbonate-rich pelagic sections, models show that erosion was limited in sites with higher clay fluxes and more likely bioturbation contributed to the lack of a short-lived excursion being preserved (*47*). Together, the transient pattern, relatively fast δ^13^C recovery, and lack of expression in pelagic records further support the argument for a short-lived POE duration on the order of centuries to millennia.

### pH magnitude and carbon released at the PETM onset

**Fig. 4.**
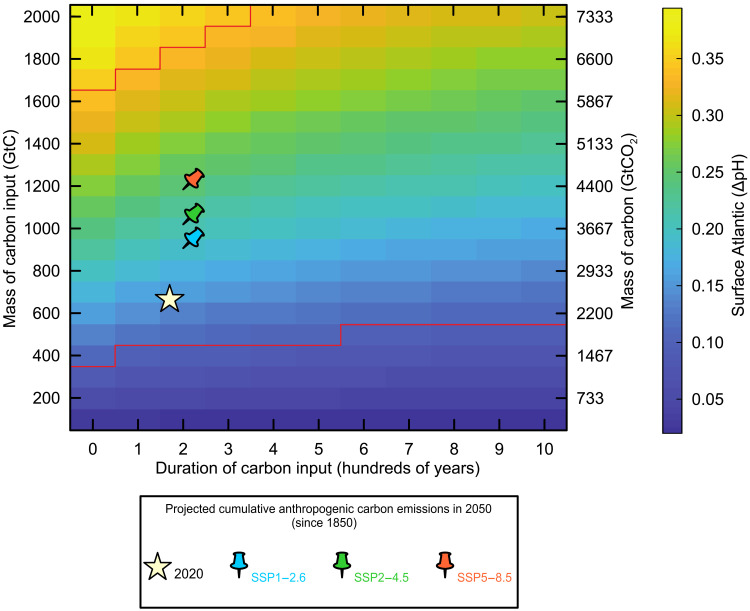
LOSCAR ΔpH output from a suite of simulations in which mass (left: *y* axis, in hundreds of Gt of carbon; right: *y* axis, in Gt of CO_2_) and duration (*x* axis, in hundreds of years) were systematically varied. The complete ΔpH output complying with a duration of carbon release from century to millennia is included. All simulations were started from equilibrium atmospheric CO_2_ of 500 ppm. The ΔpH color scale is adjusted to the limits consistent to the ΔpH (−0.08 to 0.35) based on boron isotopes with an initial surface Atlantic Ocean pH = 7.89 (table S2). Projected total anthropogenic carbon emissions relative to preindustrial (1850) in 2020 and 2050 from the SSP (shared socioeconomic pathway) scenarios (*80*) are superimposed on the LOSCAR ΔpH output array.

To further constrain the rates of carbon addition, we explore a range of carbon release scenarios using the LOSCAR (Long-term Ocean Sediment CArbon Reservoir v2.0.4), Zeebe (*30*) carbon cycle model. To estimate the potential mass of carbon released at the POE onset, we ran a suite of simulations in which the mass and duration (or rate) of carbon release was systematically varied and compared to the resulting modeled Atlantic Ocean surface acidification recorded in our δ^11^B record (Fig. 4). The magnitude of ocean pH change (ΔpH) was estimated by setting the initial pre-POE pH to the average surface Atlantic Ocean pH of 7.89 simulated by LOSCAR. The additional uncertainty of δ^11^B_sw_ on ocean pH was propagated using Monte Carlo simulation (see Materials and Methods). Given the relatively large uncertainty associated with δ^11^B-derived pH estimates (table S2), we use a conservative approach and evaluate a range of century to millennia release scenarios. To examine the surface ocean acidification response, we use modern-day mean global ocean overturning time as an upper bound for the POE carbon release duration. The lower bound estimate for the duration is currently unconstrained, and we include the full potential range from decadal to century time scale. Because the potential carbon input upper range is less constrained with the given ΔpH uncertainty, we place more confidence in our minimum carbon mass estimate (table S2). Based on an initial latest Paleocene atmospheric CO_2_ level of 500 parts per million (ppm) (*48*), a minimum ΔpH (−0.08) provides a lower estimate for the mass of carbon of ~400 Gt, and a median ΔpH (−0.35) requires a mass of carbon of ~1600 Gt, which overlaps with model estimates for the carbon released at the CIE onset (Fig. 4 and table S2) (*49*). Across the estimated POE range of carbon release mass and duration, the effects on deep-sea sedimentary CaCO_3_ are quite minor (<20%; fig. S3), records of which would be easily mixed out or dissolved by the comparatively much larger seafloor CaCO_3_ dissolution associated with the subsequent CIE, assuming that it occurred soon after the POE (<50 ka) (*47*).

Given the larger δ^13^C excursion at the main CIE (by a factor of 2) and taking the median ΔpH for the POE at face value, an isotopically heavier carbon source for the POE may be implicated. However, this simple comparison is likely complicated by the large uncertainty on our ΔpH estimate (e.g., a 2× smaller ΔpH at the POE is possible), the different durations of the carbon input for the events (*50*), and the idea that the POE most likely represents a short-lived pulse of carbon released into the atmosphere and surface ocean reservoirs. Catastrophic and near instantaneous carbon input, such as a bolide impact, does not satisfy the conditions of a common source two-phase release scenario. Impact ejecta in marine shelf records occur at or postdate the main CIE onset and do not coincide with the timing of the POE (*51*). Volcanism and contact metamorphism could provide the necessary emission of greenhouse gases, particularly CH_4_ and CO_2_, to cause the simultaneous surface ocean warming and acidification (*52*). While the emplacement of the North Atlantic Igneous Province (NAIP) generally coincides with the PETM (*53*) and recent predictive modeling suggests that NAIP carbon-based emission fluxes were sufficient to initiate warming (*52*), it remains difficult to establish a direct causal link between volcanism and climate change with the resolution of paleoclimate records. Carbon outgassing associated with the volcanic sill intrusions into organic carbon-bearing sediments is hypothesized to be the primary source of carbon for the PETM (*54*, *55*) and so could have potentially driven the POE and the main CIE. This activity could yield a wide δ^13^C input composition range (*11*) extending from mantle CO_2_ (−5‰) to thermogenic methane (−25‰) (*55*), or combinations thereof (*11*). While untestable with the current dataset, it is plausible that a short-lived C emission pulse preceded the main phase of volcanic emissions. Multiple carbon injections during the latest Paleocene could be an indication of elevated carbon cycle and climate instability. If the POE and CIE were both triggered by a common “capacitor” carbon source like methane hydrate dissociation (*56*) or permafrost thawing (*57*), it supports a positive feedback mechanism that preconditioned the Earth system to be more sensitive to subsequent perturbations, the timing of which could be set by optimized orbital configurations (*15*, *58*). An alternative hypothesis is that external volcanic forcing caused the POE with little role for amplifying feedbacks and no indication of a tipping point.

### Relevance of the POE and CIE to anthropogenic carbon emissions

**Fig. 5.**
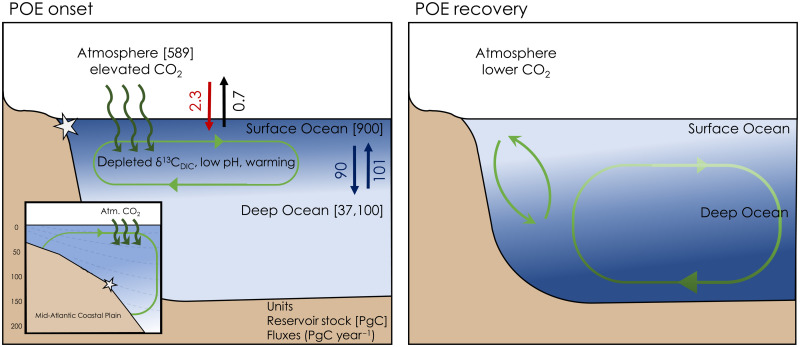
Simplified schematic of the carbon exchange between the atmosphere and ocean DIC reservoirs during the POE onset and recovery phases. Approximate preindustrial reservoir carbon stocks in PgC (1 PgC = 10^15^ gC) and annual carbon exchange fluxes are noted in parentheses (PgC year^−1^) from Ciais *et al.* (*81*). LOSCAR experiments in this study have slightly different reservoir sizes and fluxes to better represent early Paleogene ocean biogeochemistry conditions. (**Left**) The injection of depleted carbon into the atmosphere resulted in elevated CO_2_ levels, negative δ^13^C_DIC_ POE, ocean acidification, and warming. A POE duration of century to millennia limits the addition of CO_2_ and its δ^13^C signal to the surface ocean. Inset: Carbon evasion and warming are observed in coastal waters along the Mid-Atlantic Coastal Plain (SDB; star); vertical scale represents meters water depth. (**Right**) Ocean overturning circulation eventually dilutes the depleted δ^13^C and low pH surface ocean signal with the larger deep-ocean carbon reservoir.

In this study, we provide constraints on a complex scenario of carbon release before the PETM onset that allows us to reexamine its utility as a geologic analog to current anthropogenic carbon release. We provide robust evidence for a pre-CIE decline in ocean pH coupled with surface warming and associated with a CIE (POE) indicative of carbon injection before the main phase of the PETM (Figs. 2 and 3). The two carbon pulses at the POE and CIE incurred profoundly different mechanisms and associated timings of Earth system recovery following carbon release (fig. S4). The POE involved a relatively small mass of carbon (likely hundreds of GtC—similar to cumulative anthropogenic carbon emissions) released over centuries (i.e., faster or similar to ocean mixing time scales; Fig. 4), triggering a “fast” carbon cycle and climate response in which the effects were limited to the atmosphere, land surface, and ocean mixed layer, with a rapid recovery accomplished by simple mixing into the much larger deep-ocean carbon reservoir on millennial time scales (Fig. 5). The mass of carbon released during the main phase of the CIE, while similar to or larger than current fossil fuel reserves [several thousand GtC; (*11*, *49*)], was released over a considerably longer duration (several thousand to 10,000+ years; fig. S4). The slower rate of release (over a time scale longer than ocean mixing time scales) allowed for CO_2_ to be mixed into the deep ocean as it was released, which partially mitigated the severity of ocean pH decline and warming. However, once emissions stopped, the ability of the ocean and sediments to regulate calcium carbonate saturation state is overwhelmed, leaving only long-term carbon cycle feedbacks [principally silicate weathering, and also likely organic carbon burial (*59*)] to drive the recovery by removing carbon from the exogenic carbon cycle entirely. The contrast between the POE and the CIE thus offers a geologic point of comparison for the future of anthropogenic carbon emissions. If carbon emissions were to be rapidly curbed as soon as possible in our near future, the mass of C released over the last few hundred years (~2400 Gt CO_2_) would be largely removed from the atmosphere by simple mixing into the deep ocean reservoir over the next several hundred years to millennia, as it was during the recovery of the POE. However, if we proceed to release carbon at current rates for centuries until cumulative emissions approach PETM-like totals, the buffer capacity of the deep ocean to absorb carbon will be saturated, and we should expect a protracted PETM-like recovery, driven by long-term carbon cycle feedbacks over at least tens of thousands of years.

## MATERIALS AND METHODS

### Site location

The U.S. Geological Survey drilled SDB core in Talbot County, Maryland in October 2007 (Fig. 1). Lithostatigraphy, geophysical, and detailed drilling information is outlined in the preliminary report by Alemán González *et al.* (*60*). At this location, uppermost Paleocene sediments are composed of micaceous silts and glauconitic sands of the Aquia Formation and transition to lower Eocene kaolinitic clayey silt to clay of the Marlboro Clay.

### Sample preparation

The SDB core was sampled approximately every 30 cm and up to 15 cm within the CIE interval for foraminifera trace element and stable isotope analysis. Trace element and stable isotope planktonic foraminifera measurements were carried out on monogeneric samples of surface-dwelling photosymbiont-bearing *Acarinina* and *Morozovella* spp., and deeper-dwelling *Subbotina* spp. Approximately 100 to 150 specimens of the 180- to 212-μm-size fraction were used to obtain approximately 200 to 400 μg of calcium carbonate material. The gap in trace element data at the CIE onset (202.42 to 204.25 m) is due to inadequate presence of foraminifera specimens for analysis in this carbonate-poor interval. Closely spaced samples within 30 cm and a few within 150 cm of each other were often combined to achieve enough sample material for trace element analysis. Boron isotope (δ^11^B) measurements were carried out on epibenthic foraminifera *C. alleni* from the 180- to 250-μm-size fraction, and approximately 5 to 10 individuals were analyzed within each sample depth. SDB samples yield well-preserved benthic foraminifera with shell textures that do not indicate overgrowth phases or recrystallization (fig. S5). During the POE, planktonic foraminifera show evidence of postdepositional partial dissolution as indicated by removal of coarsely cancellate and muricate wall textures, resulting in smooth, thin-walled, and collapsed chambers (*23*). Poorly preserved planktonic foraminifera specimens were present in the POE interval, and only the best-preserved specimens were selected for geochemical analysis.

### δ^13^C and δ^18^O planktonic foraminiferal analyses

Stable isotope analyses were carried out on a Finnigan MAT253 mass spectrometer interfaced with a Kiel Device at University of California Santa Cruz. In this system, the sample is reacted under vacuum with phosphoric acid at 75°C, with the resulting gas distilled in a single step. Stable isotope values are reported in per mil (‰) relative to Vienna Pee Dee Belemnite standard. Analytical precision (1σ) is based on repeat analysis of an in-house standard (Carrara marble), calibrated to the international standards NBS 18 and NBS 19, and averages ±0.05‰ for δ^13^C and ± 0.08‰ for δ^18^O. Inadequate calcium carbonate (CaCO_3_) material between 204.67 and 204.34 m prevented collection for stable isotope analysis. The lack of calcium carbonate sediments at the transition into the basal section of the Marlboro Clay is commonly observed throughout the Salisbury Embayment and is interpreted as a dissolution horizon (*24*).

### Trace element foraminiferal cleaning and analysis

Trace element cleaning included clay removal, reductive, oxidative, and final acid leach steps [(*61*), later modified in (*62*)]. Foraminifera tests were gently crushed to facilitate chemical treatment, and visible infilled siliciclastic material was removed when possible. Detailed methodologies are outlined by Babila *et al.* (*25*) based on New Jersey core sites. Trace element analyses were collected on a Thermo Scientific Element XR Sector Field Inductively Coupled Plasma Mass Spectrometer at the University of California Santa Cruz. Analytical reproducibility at UCSC is 1% (2 SD) on Sr/Ca, 2% (2 SD) on Mg/Ca, and 7% (2 SD) on B/Ca based on repeated analysis of laboratory consistency standards throughout the length of the study. Samples were screened for potential contamination of clay and diagenetic iron bearing mineral phases, and no correlation was observed between Mg/Ca and B/Ca with Al/Ca, Mn/Ca, or Fe/Ca. Average planktonic foraminifera yielded Al/Ca values of 22 ± 21 μmol/mol (SD), Mn/Ca values of 306 ± 203 μmol/mol (SD), and Fe/Ca values of 1424 ± 1035 μmol/mol (SD). The presence of secondary calcite was monitored analytically using Sr/Ca as an indicator of diagenetic carbonate based on the understanding that recrystallized calcite contains less Sr (*63*, *64*). Planktonic foraminiferal Sr/Ca values at SDB were greater than the threshold value of 1.0 mmol/mol (*63*), with the exception of three sample depths near the dissolution horizon that were excluded in the reconstructions.

### δ^11^B laser ablation analytical protocol

Benthic foraminiferal boron isotopes (δ^11^B) and B/Ca (μmol/mol) were collected by laser ablation multicollector inductively coupled plasma–mass spectrometry (LA-MC-ICP-MS), which was performed at the University of Southampton using a Thermo Scientific Neptune Plus MC-ICP mass spectrometer (Thermo Fisher Scientific, Waltham, MA, USA) coupled to an Elemental Scientific Lasers (Bozeman, MT, USA) New Wave Research 193 excimer laser ablation system with a TwoVol2 ablation chamber. Before LA-MC-ICP-MS analysis, foraminifera were cleaned following a treatment adopted from previous solution MC-ICP-MS protocols (*28*, *65*) to include clay removal and oxidative steps but omitting the final acid leach. Benthic foraminifera were cleaned individually as whole tests to maintain chamber orientation and to ensure test integrity during ablation. Cleaned specimens were mounted on double-sided tape, with the umbilical side facing upward.

Analysis broadly followed protocols published by Standish *et al.* (*29*). Boron isotopes were measured on the L3 and H3 Faraday cups installed with 10^13^-ohm resistors, except for Paleocene Bass River samples that used 10^12^-ohm resistors. Before data collection, standards were ablated to remove any surface contamination using the following settings: 5 Hz repetition rate, 100 μm s^−1^ laser tracking speed, and ~5 J cm^−2^ laser power density. Foraminiferal test surfaces were not cleaned by ablation before analyses due to their fragility; cleaning relied on the chemical procedures outlined above, and analysis of time-resolved data suggested that negligible surface contamination was present. Data were collected in static mode using integrations of 2.194 s. Analyses of reference materials integrate 100 cycles, and the number of cycles analyzed for foraminifera varied based on test size. Dynamic blank corrections were applied cycle-by-cycle assuming a linear relationship between the preceding and succeeding blank measurements. Instrumental mass bias was corrected by sample-standard bracketing with glass reference material NIST SRM610 with an isotopic ratio of ^11^B/^10^B = 4.049 and composition of δ^11^B = −0.26‰ (*29*, *66*). Matrix interference from scattered calcium ions on ^10^B were corrected using the log-relationship between δ^11^B inaccuracy and ^11^B/Ca_interference_ based on well-characterized δ^11^B and B/Ca published values for carbonate reference materials. Synthetic carbonate pellet MAC-3 δ^11^B value is −0.57 ± 0.11‰ (2 SD) and B/Ca value is 56 ± 2 μmol/mol (2 SD) analyzed by Standish *et al.* (*29*). *Porites* sp. coral JCp-1δ^11^B value used is 24.36 ± 0.45‰ (2 SD), and biogenic aragonite *Tridacna gigas* JCt-1 value used is 16.39 ± 0.60‰ (2 SD), determined in the interlaboratory comparison of Gutjahr *et al.* (*67*). Preferred published B/Ca ratio for JCp-1 is 460 ± 23 (SD) and that for JCt-1 is 191 ± 9 (SD), collated by Hathorne *et al.* 2013 (*68*). Ca_interference_ was measured at mass 10.10 using the L2 Faraday cup (installed with a 10^12^-ohm resistor). Benthic foraminifera B/Ca values for the cleaning experiments (figs. S6 and S7) were calculated by normalizing the measured ^11^B/Ca_interference_ of the sample to carbonate reference standard JCt-1 with a value of 191 ± 9.3 μmol/mol (2 SD) (*68*), as detailed by Standish *et al.* (*29*). Laser ablation tracks on benthic foraminifera were carried out as a line raster in an approximate spiral pattern centered across the chambers of the final coil on the umbilical side. The spot size for each foraminifer individual was adjusted from 40 to 150 μm to ensure that the laser ablation track was completely overlaid on the whole specimen. A total of three passes of the set track on each benthic foraminifer individual were carried out. The nonuniform test wall thickness of the sample individuals resulted in variable amount of test material being ablated during a given measurement session. When the test wall was ablated through, we used the ^11^B and Ca intensities to screen for the lack of foraminiferal material and omitted those data from the final analysis. Cycles falling outside of the 2 SD of the mean were omitted. δ^11^B data were screened on several analytical criteria to be considered suitable for pH reconstruction: Only δ^11^B analyses with a ^11^B intensity >20 mV, a blank correction <10%, and with enough cycles to give an internal precision <1.2‰ were used. The precision cutoff of <1.2‰ was used as an indicator for a poor analysis composed of loss of signal or was short analysis duration. For context, the average precision at 2 SE for the data shown in figs. S6 and S7 was ~0.7‰. A total of 40 δ^11^B *C. alleni* measurements did not meet the above criteria and were not included in the pH reconstruction. For each depth sampled, the acceptable LA-derived δ^11^B measurements were averaged and a 2 SE uncertainty was calculated. In addition to the suite of carbonate reference materials (e.g., JCp-1, JCt-1, and MACS-3), the internal reference material PS69/318-1 composed of a fragment of deep-sea coral bracketed samples was analyzed throughout all analytical sessions. The long-term mean was based on repeat analyses of PS69/318-1 (total = 35) during six analytical sessions spanning the duration of the study (2019) to yield a δ^11^B value of 14.15 ± 0.79‰ (2 SD) and a B/Ca value of 188.57 ± 44.91 μmol/mol (2 SD). The long-term mean determined by laser ablation is consistent with solution MC-ICP-MS and ICP-MS measurements of 13.83 ± 0.29‰ (2 SE) and 198 ± 7.9 μmol/mol, respectively [(*29*) for δ^11^B and (*28*) for trace elements]. The final uncertainty applied to our laser δ^11^B data ranged from 0.9 to 1.3‰ (at 95% confidence) and was determined by a quadratic addition of the 2 SE associated with the mean for each depth and the uncertainty in our internal reference material PS69/318-1.

In light of the variability that exists in single specimen δ^11^B [this study; Raitzsch *et al.* (*69*)], and given that the number of individual analyses comprising an average value here ranges from 3 to 8, we have investigated how representative the means we calculate are. To do this, we took the cleaning test data discussed below (figs. S6 and S7), removed the untreated samples, and converted each time interval into a relative δ^11^B value by subtracting its mean. The SD of this dataset of *n* = 38 was 1.0‰. A normal distribution with a mean = 0 and SD = 1.0 was generated and this was repeatedly sampled (*n* = 1000), and averages were calculated using 3 to 8 values. This treatment reveals that there is a 95% chance that a laser δ^11^B mean consisting of three measurements is within 1.2‰ of the true value; for eight measurements, there is a 95% chance it lies within 0.8‰ of the true value. Given that our uncertainties on our δ^11^B averages range from 0.9 to 1.3‰, we can be confident that the true value is encompassed by our uncertainty interval.

### δ^11^B foraminifera cleaning assessment

To assess potential postdepositional contamination on δ^11^B and B/Ca measurements, several cleaning tests were conducted on *C. alleni* from nearby ODP Bass River where specimens are more abundant than at SDB and are present across the Paleocene-Eocene boundary (Fig. 1). Well-preserved Early Pleistocene *Cibicidoides wuellerstorfi* specimens from ODP Site 999 were also measured as a comparative reference (fig. S6). Subsets of individuals were analyzed for δ^11^B, untreated either with the clay removal step only or with the full treatment that includes both clay and oxidative steps. *C. wuellerstorfi* ODP Site 999 specimens analyzed untreated yielded a mean δ^11^B value of 15.76 ± 1.0‰ (2 SE) and a B/Ca value of 180.08 ± 54 μmol/mol (2 SE; fig. S6). *C. wuellerstorfi* ODP Site 999 specimens subjected to the full cleaning protocol yielded a mean δ^11^B value of 15.66 ± 0.8‰ (2 SE) and a B/Ca value of 179.02 ± 48 μmol/mol (2 SE) and are statistically indistinguishable from measured untreated specimen δ^11^B and B/Ca values (fig. S6). At Bass River, benthic foraminifera in the uppermost Paleocene glauconitic sands of the Vincentown Formation are fairly well preserved but are frosty in appearance, and some are broken or abraded (*22*). *C. alleni* Bass River Paleocene specimens analyzed after the clay removal step yielded a mean δ^11^B value of 12.71 ± 1.1‰ (2 SE) and B/Ca value of 140.26 ± 47 μmol/mol (2 SE), and specimens subjected to full cleaning protocol yielded a mean δ^11^B value of 13.40 ± 1.2‰ (2 SE) and a B/Ca value of 127.93 ± 46 μmol/mol (2 SE; fig. S7). Specimens in the clay-rich Marlboro Clay are excellently preserved (translucent appearance) and not abraded (*22*). *C. alleni* Bass River Eocene specimens analyzed untreated yielded a mean δ^11^B value of 11.93 ± 1.1‰ (2 SE) and a B/Ca value of 126.74 ± 51 μmol/mol (2 SE), and specimens subjected to the full cleaning protocol yielded a mean δ^11^B value of 13.02 ± 1.0‰ (2 SE) and a B/Ca value of 108.18 ± 45 μmol/mol (2 SE; fig. S7). Despite the distinct lithologies and therein diagenetic histories of specimens obtained from the siliciclastic Vincentown Formation (Paleocene) and clay-rich Marlboro Clay (Eocene), δ^11^B and B/Ca values were not changed between cleaning procedures within the analytical uncertainty (fig. S7).

Surficial cleaning of foraminiferal tests does not preclude possible chamber infilling and partial diagenetic recrystallization. To test this issue further, the exact samples analyzed for δ^11^B were also analyzed for trace elemental composition by laser ablation quadrupole ICP-MS at the University of Southampton. Elements indicative of clay (Al) and metal oxide (Fe and Mn) minerals were substantially lower in fully cleaned specimens compared to those that were untreated. No discernible relationship was observed between Al, Mn, and Fe versus δ^11^B, suggesting that, if noncarbonate phases were present, they are insufficient to alter the δ^11^B away from primary test values. Because of the limited sample material, elemental screening was not possible on all samples before δ^11^B analysis, and as a precaution, all samples used in pH reconstructions underwent full cleaning treatment.

### Mg/Ca-derived ocean temperatures

In addition to temperature, nonthermal controls are known to influence magnesium incorporation into foraminiferal tests. Mainly through laboratory culture experiments, these secondary controls are recognized as salinity (*70*), carbonate chemistry (pH or carbonate ion) (*71*), and secular variation in seawater Mg/Ca (Mg/Ca_sw_) for deep-time reconstructions (*72*). Late Paleocene Mid-Atlantic coastal surface salinity values are estimated to be approximately 35 based on an average for the upper 200 m in the North Atlantic Ocean in the PETM coastal model simulation of Hantsoo *et al.* (*73*). Salinity variations over the PETM at nearby New Jersey sites are computed to be less than 3 units (*6*, *26*) and therefore contribute an uncertainty of ~1°C on Mg/Ca-derived temperatures. Compiled PETM (*6*) and POE boron isotope pH records were used to correct measured Mg/Ca ratios with the Mg/Ca-pH relationship derived from modern planktonic foraminifera (*74*). Mid-Atlantic foraminiferal δ^11^B measurements are of limited resolution to permit a paired Mg/Ca pH correction scheme. Instead, the pre-POE pH value is fixed and ΔpH reconstructions here and globally (*6*, *11*, *17*, *27*) at the POE and CIE were used to interpolate for Mg/Ca without a paired pH value. Carbon isotope records presented here and biostratigraphy zonation documented by Self-Trail *et al.* (*19*) were used to determine the following depth intervals: pre-POE (below 207.1 m), POE (207.1 to 205.9 m), pre-CIE (205.9 to 204 m), CIE (204 to 197.8 m), CIE recovery phases I (197.8 to 190.5 m) and II (190.5 to 187.1 m), and post-CIE (above 187.1 m). A fixed late Paleocene pH = 7.89 was used to correct Mg/Ca values in the pre-POE, pre-CIE, and post-CIE intervals. Based on the median ΔpH anomaly at the POE of −0.35, a pH = 7.54 was applied to correct Mg/Ca values within the POE. Based on a ΔpH anomaly at the PETM of −0.4 (*6*), a pH = 7.49 was applied to correct Mg/Ca values within the CIE. During CIE recovery phases I and II, the pH was increased incrementally (e.g., 7.6 and 7.7, respectively) toward the baseline late Paleocene pH. To convert measured planktonic foraminifera Mg/Ca into temperature, we first corrected for the evolution of seawater pH change at SDB using the Mg/Ca-pH relationship in (*74*). pH-normalized Mg/Ca values were converted to temperature using the exponential equation form Mg/Ca_foraminifera_ = *B* exp*^AT^*, where *T* is the temperature (°C), *B* is the preexponential constant, and *A* is the exponential constant, which vary as a function of Mg/Ca_sw_. The nonlinear relationship between Mg/Ca_sw_ and foraminiferal Mg/Ca requires modified coefficients to account for varying temperature sensitivity between modern and Paleogene Mg/Ca_sw_ values (*48*, *72*). Modern symbiont-bearing planktonic foraminifera *Globigerinoides ruber* Mg/Ca calibration is the only available study to consider a nonlinear correction under variable Mg/Ca_sw_ for planktonic foraminifera and was used to compute pH-normalized Mg/Ca into temperature (*75*). Calcium and magnesium concentrations are assumed to be constant over the studied interval, as their oceanic residence times are considerably longer than the entire duration of the PETM (*76*). Early Paleogene Mg/Ca seawater reconstructions are generally limited to model estimates with large discrepancies among records. Evans *et al.*’s (*77*) geochemical approach paired clumped isotopes to Mg/Ca to reconstruct Mg/Ca_sw_ over the Paleogene and were able to generate Eocene temperatures consistent with model simulations. For these reasons, we consider a PETM (~56 Ma) Mg/Ca_sw_ value of 2.50 (range, 0.32 to 0.41 mmol/mol) (*77*) to be more appropriate than model-based reconstructions over the same time interval. Based on the preferred PETM Mg/Ca_sw_ reconstructed value, the average preexponential constant B calculated is 0.52 (range, 0.50 to 0.55) and exponential constant A is 0.06 (range, 0.05 to 0.07).

### δ^11^B-derived seawater pH

The magnitude and spatial pattern of ocean acidification during the PETM was previously derived using boron isotopes in planktonic foraminifera (*6*, *11*, *17*, *27*). The SDB core is dominated by siliciclastic sediments, and planktonic foraminifera are sparse in the studied late Paleocene interval and generally limited to smaller specimens (<250 μm). The limited planktonic foraminifera content is insufficient to analyze δ^11^B by traditional solution methods (*6*, *27*). Larger-sized (250 to 500 μm) benthic foraminifera are sufficiently abundant to yield pH records that are both continuous and of similar resolution to complementary geochemical records during the POE interval when analyzed by laser ablation approaches. Deep-time reconstruction of ocean pH based on foraminifera δ^11^B requires both the knowledge of species-specific δ^11^B-pH calibrations and past seawater δ^11^B (δ^11^B_sw_) composition. In addition, temperature and salinity are needed to estimate the dissociation constant of boric acid (K^*^_B_). No correction for the effect of secular seawater Mg and Ca concentration variation on pK^*^_B_ was applied. When the pre-POE pH is fixed, the choice whether to correct pK*_B_ variable seawater composition makes little impact on the estimated magnitude of pH change. Modern benthic foraminifera core-top calibrations document species-specific δ^11^B_foram_ to δ^11^$B_{B{\left(\text{OH}\right)}_{4}^{-}}$ relationships that are variably offset from seawater borate (δ^11^$B_{B{\left(\text{OH}\right)}_{4}^{-}}$) values (*65*). δ^11^B values of *Cibicidoides* and other epibenthic species are within predicted equilibrium seawater δ^11^$B_{B{\left(\text{OH}\right)}_{4}^{-}}$ values, and no vital effect is observed (δ^11^B_foram_ = δ^11^$B_{B{\left(\text{OH}\right)}_{4}^{-}}$). Based on the assumption that Paleogene *C. alleni* δ^11^B systematics are analogous to modern *Cibicidoides*, no isotopic offset was applied to derive ocean pH. The assumption that δ^11^B of *Cibicidoides* spp. reflects seawater δ^11^$B_{B{\left(\text{OH}\right)}_{4}^{-}}$ is corroborated by model-data depth profile comparisons throughout the Eocene epoch (*78*).

To evaluate the uncertainty of Paleogene δ^11^B_sw_ composition on the determination of ocean pH, a similar Monte Carlo approach used by Gutjahr *et al.* (*11*) was adopted. Estimation of background Paleocene hydrographic parameters (e.g., temperature and salinity) was based on a Regional Ocean Modeling System (ROMS) simulation of shelf conditions within the Salisbury Embayment (*73*). Following the approach of previous PETM boron isotope studies, pre-POE δ^11^B values were normalized to a fixed surface ocean pH value because of the uncertainty associated with δ^11^B_sw_ (*6*, *11*, *17*, *27*). The higher the assumed pre-POE pH, the lower δ^11^B_sw_ value is required and the smaller pH magnitude (ΔpH) is recorded (fig. S8). Based on the ROMS Mid-Atlantic coastal modeling (*73*), we assume an average Paleocene shelf bottom water temperature of 22°C, salinity equal to 35, and pH of 7.67. Using this hydrographic information, we set pre-POE δ^11^B values to pH = 7.67, which, when coupled with our measured δ^11^B, required a δ^11^B_sw_ = 37.8 ± 0.46‰. The uncertainty in δ^11^B_sw_ is characterized by 10,000 realizations that translated δ^11^B to pH by randomly varying δ^11^B within 2σ analytical uncertainty, temperature by ±2°C, and salinity by ±1 unit. Although this is close to the δ^11^B_sw_ estimate of Gutjahr *et al.* (*11*) using a similar approach (37.6 ± 0.5‰ to 38.94 ± 0.41‰), the above conditions resulted in failures of 4614 of the 50,000 realizations (10,000 per sample depth) because pH values were incomputable, as δ^11^B was below what is possible given an isotopic fractionation of 27.2‰ (*79*). Ultimately, to be consistent with LOSCAR 500 ppm CO_2_ simulation, a pre-POE surface Atlantic Ocean pH of 7.89 was applied. A pre-POE fixed of pH = 7.89 required a δ^11^B_sw_ = 36.3 ± 0.48‰ and resulted in failures of 118 of 50,000 realizations (10,000 per sample depth). The higher initial pH is also favored as it resulted in fewer model failures. In the absence of improved estimates of late Paleocene to early Eocene δ^11^B_sw_, it is currently not possible to refine our estimates of ΔpH further.

### LOSCAR carbon cycle modeling

LOSCAR v2.0.4 was used in the Paleogene configuration (including a Tethys ocean) (*30*). The model was spun up to long-term carbon cycle equilibrium (with balanced fluxes for all tracers) before carbon release scenarios. Results in the main text use an equilibrium CO_2_ (the CO_2_ at which carbon input via solid-earth degassing is perfectly balanced by silicate weathering and carbonate burial) of 500 ppm. A simple climate sensitivity of 4.5°C per CO_2_ doubling (relative to the equilibrium CO_2_) was used. Each carbon release simulation was run for 10 ka, with carbon release beginning at the start of each run. The LOSCAR delta-pH array (Fig. 4) is the minimum surface Atlantic pH (which occurs shortly after the carbon release is complete) minus initial surface Atlantic pH.
