# Supplementary material for: Surface ocean warming and acidification driven by rapid carbon release precedes Paleocene-Eocene Thermal Maximum
Source: Sci Adv. 2022 Mar 16;8(11):eabg1025. doi: 10.1126/sciadv.abg1025 (PMC8926327; doi:10.1126/sciadv.abg1025)
Supplement: Supplementary file 1 — Tables S1 and S2 Figs. S1 to S8 [file sciadv.abg1025_sm.pdf]

Science Advances
AAAS

Supplementary Materials for

Surface ocean warming and acidification driven by rapid carbon release precedes Paleocene-Eocene Thermal Maximum

Tali L. Babila*, Donald E. Penman, Christopher D. Standish, Monika Doubrawa, Timothy J. Bralower, Marci M. Robinson, Jean M. Self-Trail, Robert P. Speijer, Peter Stassen, Gavin L. Foster, James C. Zachos

*Corresponding author. Email: t.babila@soton.ac.uk

Published 16 March 2022, *Sci. Adv.* **8**, eabg1025 (2022)
DOI: 10.1126/sciadv.abg1025

The PDF file includes:

Tables S1 and S2
Figs. S1 to S8

Other Supplementary Material for this manuscript includes the following:

Data S1

Table S1.

Estimation of the POE duration and time between the onset of the POE and the main CIE from the South Dover Bridge core using regional sedimentation rates estimated by linear accumulation rates. Uppermost Paleocene sedimentation rates (cm/kyr) used in the computations are from South Dover Bridge and additional sites located within the Salisbury Embayment.

Site	Reference	Average sedimentation rate at	POE duration	Time between POE to CIE
Wilson Lake	1	0.1 cm/kyr	1070 kyr	1740 kyr
	2	8.4 cm/kyr	12.7 kyr	20.7 kyr
Bass River	1	1.0 cm/kyr	107 kyr	174 kyr
	2	2.5 cm/kyr	42.8 kyr	69.6 kyr
South Dover Bridge	3	0.5 cm/kyr	214 kyr	348 kyr
South Dover Bridge*	4	19.5 cm/kyr	5.5 kyr	8.9 kyr
	4	53.5 cm/kyr	2.0 kyr	3.3 kyr

[1]Stassen *et al.* (*42*), [2]John *et al.* (*31*), [3]Lyons *et al.* (*39*), and [4]Bowen *et al.* (*18*).

***** Calculation of the time between the onset of the POE and the main CIE based on Big Horn Basin used a fixed POE duration of 2 and 5.5 kyr from Bowen *et al.* (*18*) to estimate the average South Dover Bridge sedimentation rate (cm/kyr).

Table S2.

Magnitude of ocean pH change (ΔpH) at the POE based on *Cibicidoides alleni* boron isotope record at South Dover Bridge. The uncertainty estimates at varying confidence intervals (%) of ΔpH was assessed using a Monte Carlo approach based on varying baseline Paleocene pH value and Paleogene $\delta^{11}B_{sw}$ composition. ΔpH reported is the difference between the fixed pre-POE pH value and average of pH values within the POE.

Paleocene pH= 7.67					
	**2.5%**	**14.0%**	**50.0%**	**86.0%**	**97.5%**
**ΔpH**	-1.74	-1.04	-0.54	-0.25	-0.08

Paleocene pH= 7.89					
	**2.5%**	**14.0%**	**50.0%**	**86.0%**	**97.5%**
**ΔpH**	-0.96	-0.59	-0.35	-0.18	-0.08

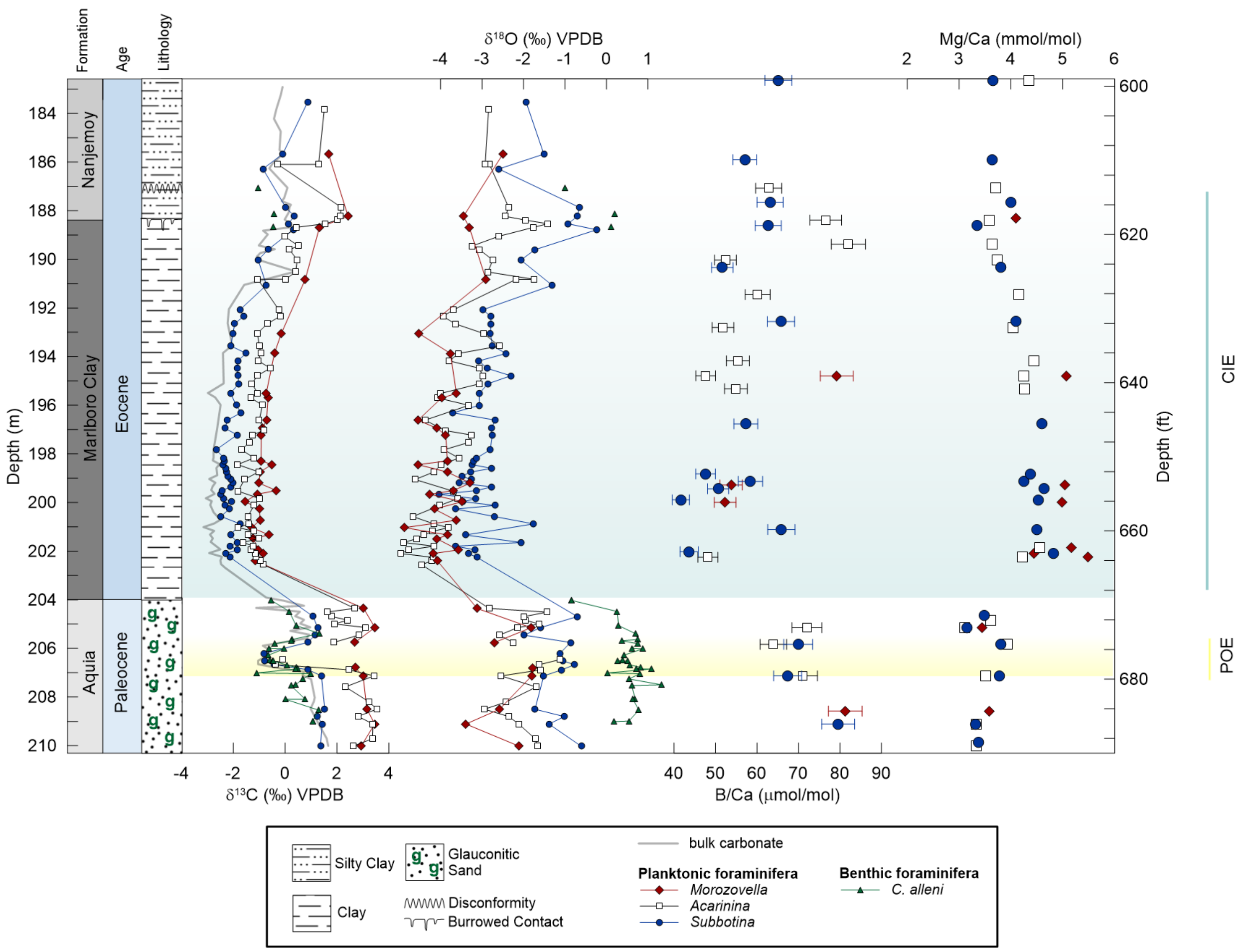

Figure S1. South Dover Bridge stable isotope bulk carbonate (*19*), planktonic and benthic foraminifera records. Benthic foraminifera stable isotope ($\delta^{13}C$ and $\delta^{18}O$) records are based on *Cibicidoides alleni*. Planktonic foraminifera B/Ca µmol/mol and Mg/Ca mmol/mol are based on monogeneric multi-specimen analysis of *Morozovella*, *Acarinina* and *Subbotina*. The upper Paleocene Aquia Formation is overlain by the lowermost Eocene Marlboro Clay and Nanjemoy Formation. Core lithology is comprised of glauconitic sands, silty clay and clay units. Highlighted in yellow is the pre-onset excursion (POE) and in blue is the body of the carbon isotope excursion (CIE).

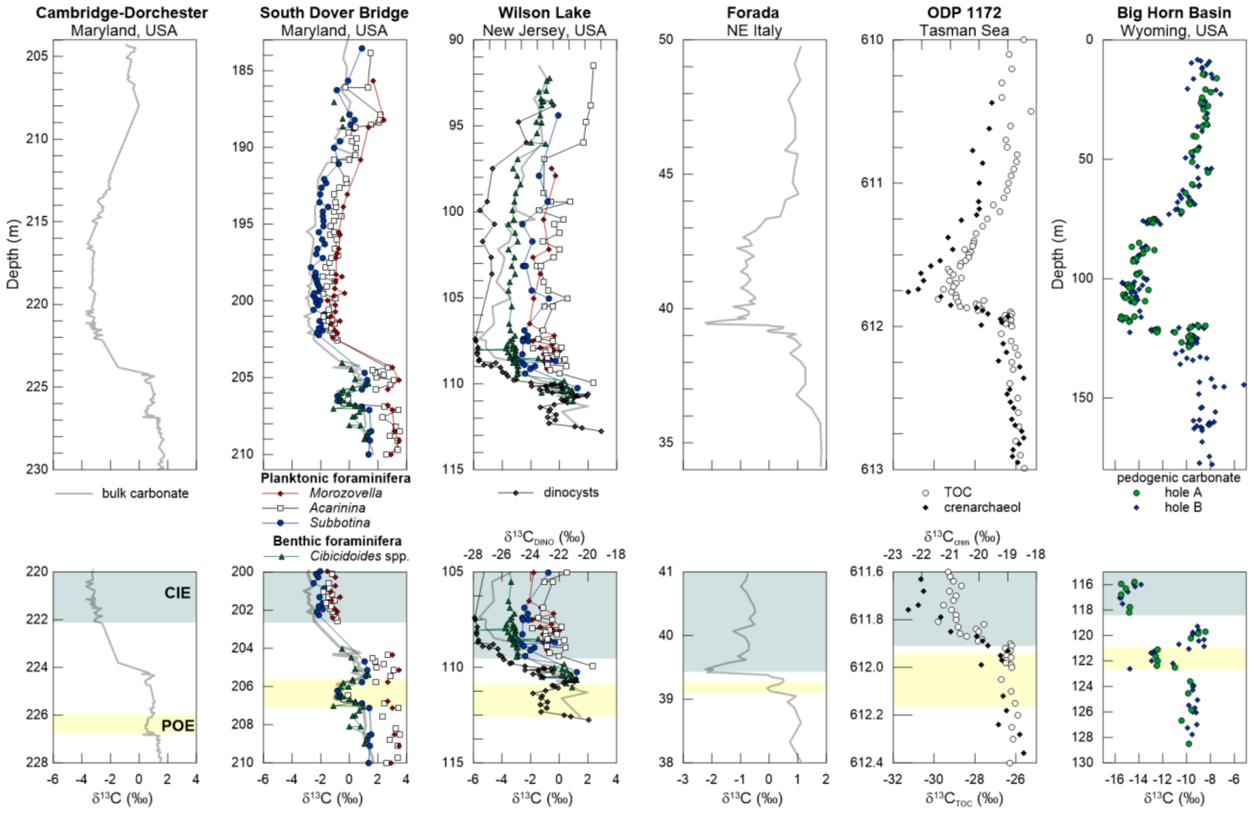

Figure S2. Marine and terrestrial carbon isotope ($\delta^{13}C$) records across the Paleocene-Eocene Thermal Maximum at six globally distributed sites. $\delta^{13}C$ bulk carbonate data are from CamDor (*39*), SDB (*19*), Wilson Lake (*26*) and Forada, Italy (*20*). $\delta^{13}C$ records at IODP Site 1172, Tasman Sea (*21*) are based on bulk total organic carbon ($\delta^{13}C_{TOC}$) and crenarchaeol ($\delta^{13}C_{cren}$). The Big Horn Basin $\delta^{13}C$ core records were generated from pedogenic carbonates (*18*). $\delta^{13}C$ records at Wilson Lake are based on planktonic and benthic foraminiferal data (*26*, *42*) and dinocysts (*13*). Planktonic and benthic foraminiferal $\delta^{13}C$ records at SDB were generated in this study. The pre-onset excursion (POE) is highlighted in yellow, and the carbon isotope excursion (CIE) is highlighted in blue. The excursion base is placed at the $\delta^{13}C$ minimum value.

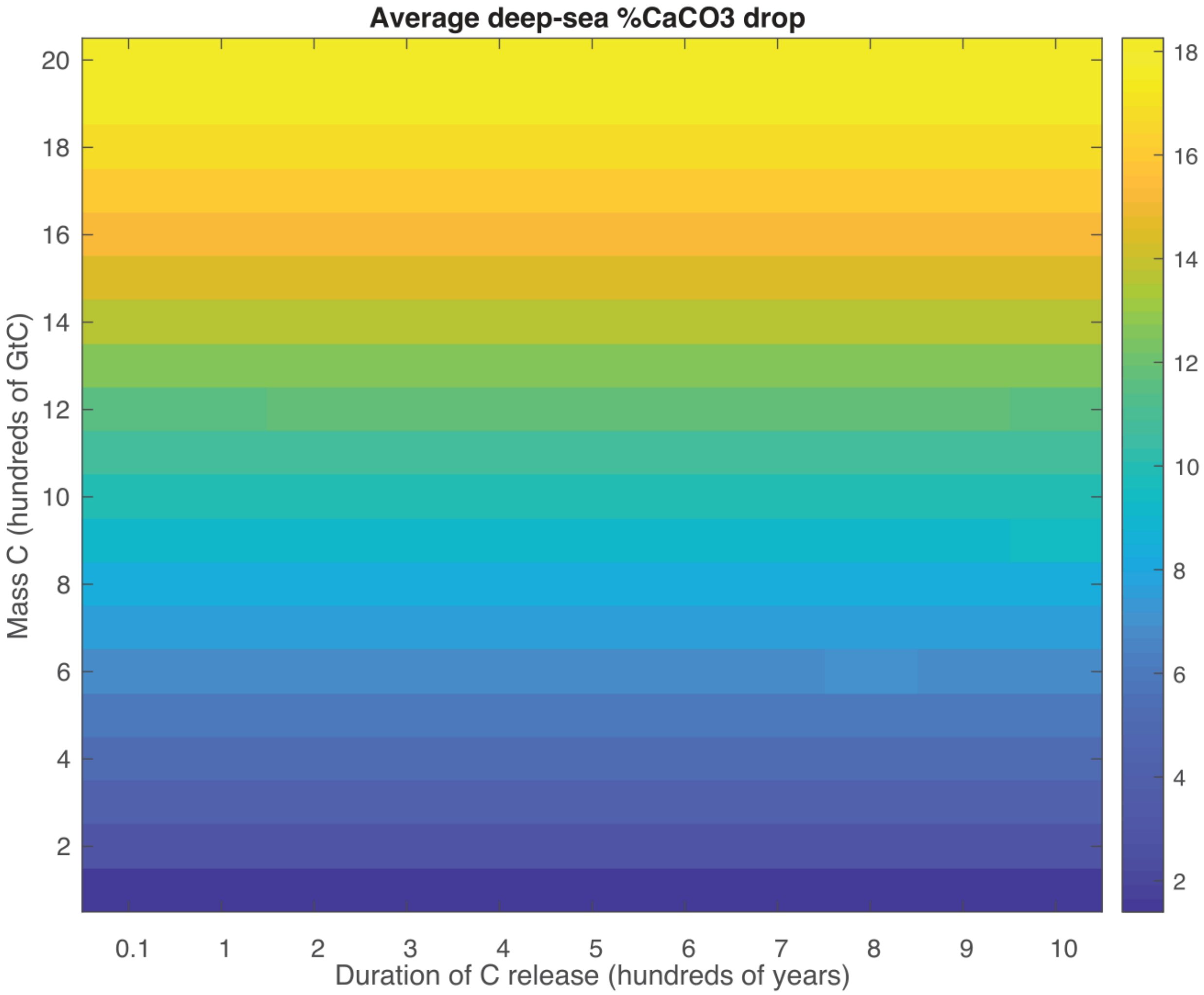

Figure S3. LOSCAR average deep-sea percent calcium carbonate ($CaCO_3$) decline as a function of the mass (y-axis, in hundreds of Gt of carbon) and duration (x-axis, in hundreds of years) were systematically varied.

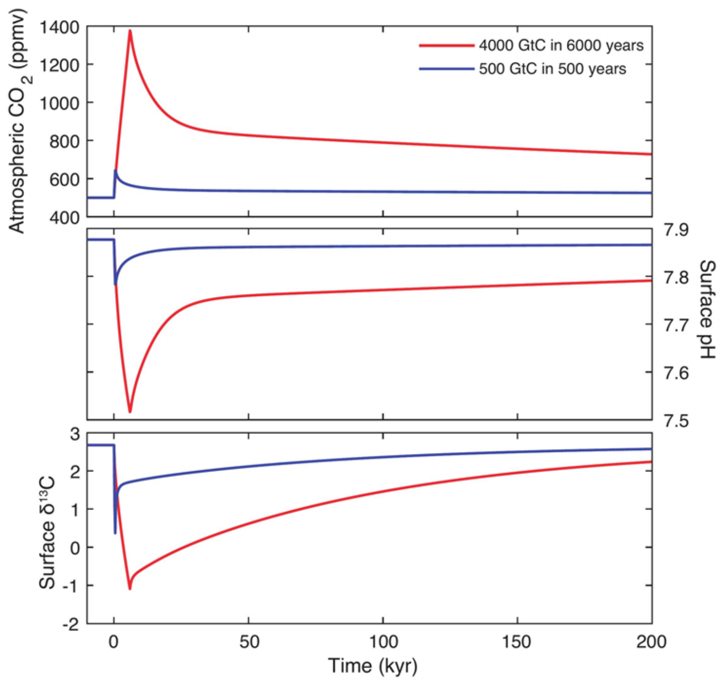

Figure S4. LOSCAR records of atmospheric CO_2 (ppmV) and Atlantic Ocean surface pH and $\delta^{13}C$ composition following the carbon release. Two scenarios are presented to simulate carbon release similar to the POE and CIE events. For a short-lived POE-like scenario, a carbon release rate of 500 Gt in 500 years was applied ($\delta^{13}C$ = -20‰), and for a longer-lived CIE-like scenario, a carbon release rate of 4000 Gt in 6000 years ($\delta^{13}C$ = -55‰) was applied. The carbon isotopic composition was modified to approximate the mean magnitude of the $\delta^{13}C$ excursion of POE and CIE marine records. Note the following scenarios are not meant to exactly reproduce either event but to demonstrate the relationship between the duration of a carbon release and the relative recovery time.

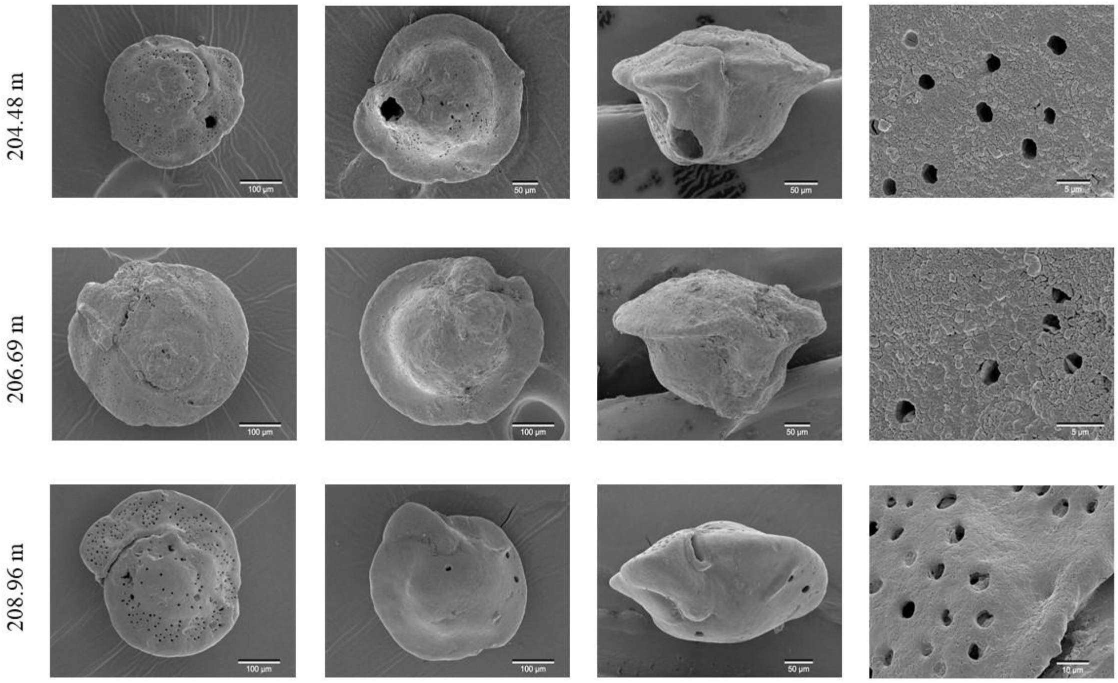

Figure S5. Scanning electron microscope (SEM) images of benthic foraminifera *Cibicidoides alleni* specimens at discrete sample depths over the pre-onset excursion (POE) from South Dover Bridge: 208.96 m (pre-POE), 206.69 m (POE) and 204.48 m (post-POE).

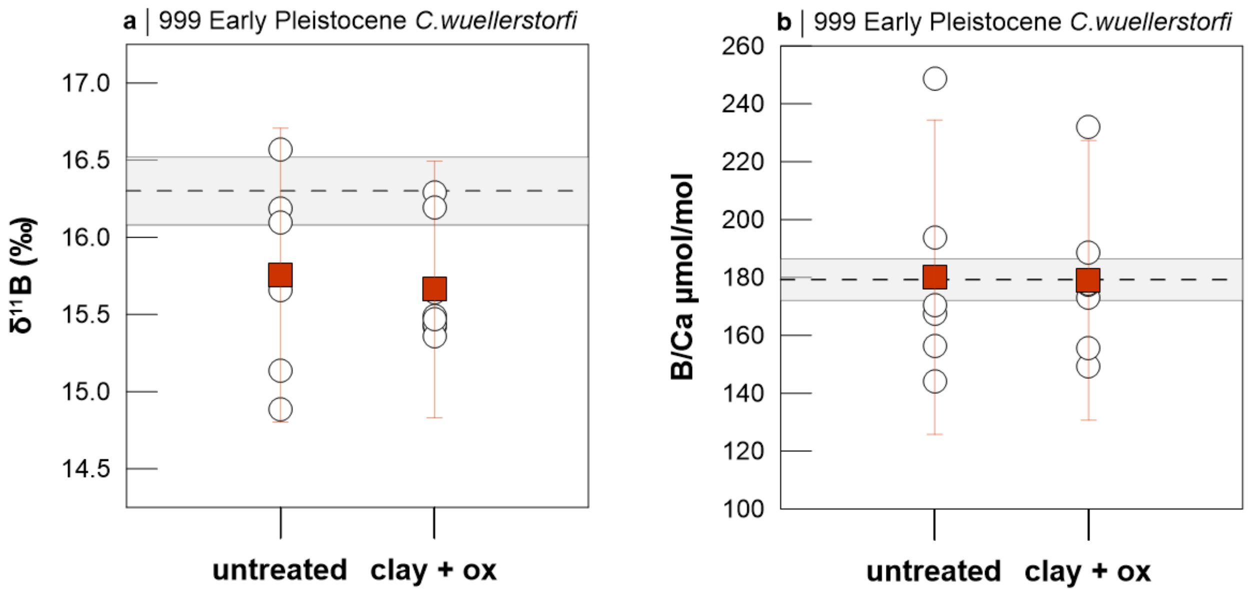

Figure S6. Early Pleistocene *Cibicidoides wuellerstorfi* (a) $\delta^{11}B$ (‰) and (b) B/Ca (µmol/mol) from ODP Site 999 of single specimens analyzed untreated and with clay removal and oxidative step completed. Open symbols are individual measurements and the average is represented by a solid symbol with 2SE associated with the mean for each depth and the uncertainty in our internal reference material PS69/318-1. Complementary solution MC-ICP-MS $\delta^{11}B$ value of 16.30 ± 0.22‰ and an ICP-MS B/Ca value of 179.27 ± 5 % are compared to mean laser ablation analysis.

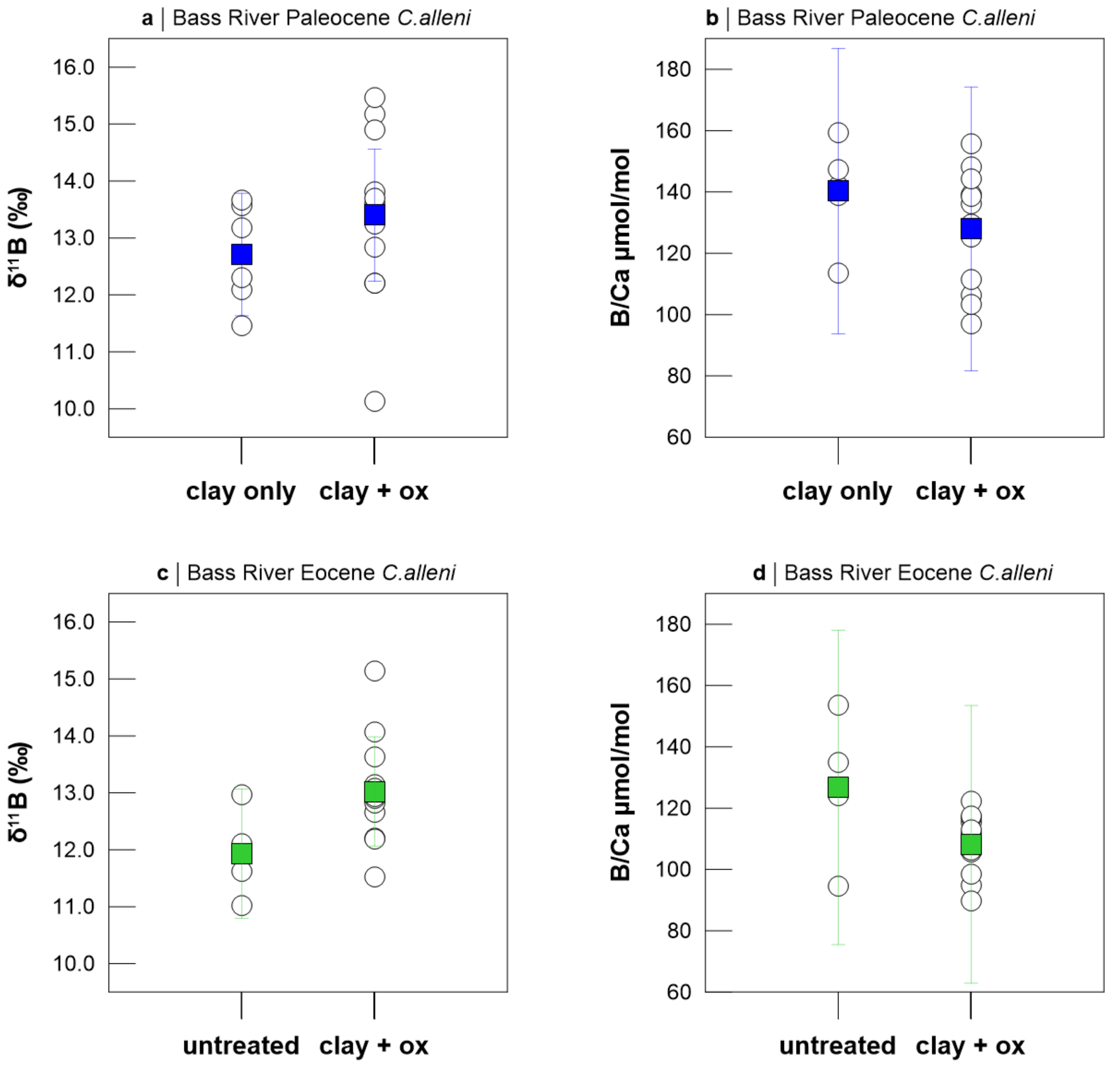

Figure S7. $\delta^{11}B$ (‰) and B/Ca (µmol/mol) measured on uppermost Paleocene (a, b) and lowermost Eocene (c, d) *Cibicidoides alleni* from ODP Bass River of single specimens analyzed untreated, clay removal only and with clay removal and oxidative step completed. Open symbols are individual measurements, and the average is represented by a solid symbol with 2SE associated with the mean for each depth and the uncertainty in our internal reference material PS69/318-1.

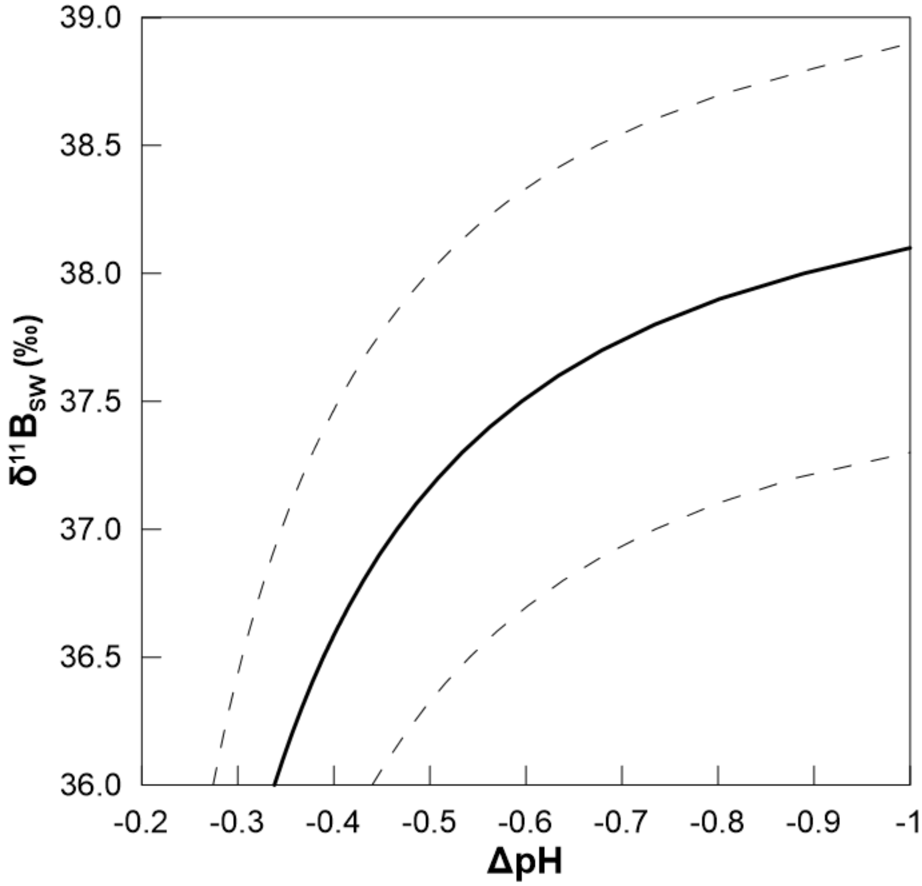

Figure S8. Boron isotope ($\delta^{11}B$) proxy sensitivity test for benthic foraminifera (*Cibicidoides alleni*) in the latest Paleocene at SDB. The magnitude of ocean pH (ΔpH) at the POE is derived for a range of boron isotopic seawater values ($\delta^{11}B_{SW}$) based on constant salinity (S=35) and a 2°C temperature change ($T_{initial}$ = 22°C). Ocean pH is computed by assuming $\delta^{11}B_{foram} = \delta^{11}B_{B(OH)_4^-}$ and no vital effect is applied.
